# Intrathecal Immunoselective Nanopheresis for Alzheimer’s Disease: What and How? Why and When?

**DOI:** 10.3390/ijms251910632

**Published:** 2024-10-02

**Authors:** Manuel Menendez-Gonzalez

**Affiliations:** 1 Departamento de Medicina, Facultad de Ciencias de la Salud, Universidad de Oviedo, ES-33006 Oviedo, Spain; menendezgmanuel@uniovi.es; 2 Hospital Universitario Central de Asturias, Servicio de Neurología, ES-33011 Oviedo, Spain; 3 Instituto de Investigación Sanitaria del Principado de Asturias (ISPA), ES-33011 Oviedo, Spain

**Keywords:** intrathecal delivery, immunoselective nanopheresis, neurodegenerative diseases, monoclonal antibodies, nanoporous membranes, drug delivery systems, amyloid-oligomers, Alzheimer’s disease

## Abstract

Nanotechnology is transforming therapeutics for brain disorders, especially in developing drug delivery systems. Intrathecal immunoselective nanopheresis with soluble monoclonal antibodies represents an innovative approach in the realm of drug delivery systems for Central Nervous System conditions, especially for targeting soluble beta-amyloid in Alzheimer’s disease. This review delves into the concept of intrathecal immunoselective nanopheresis. It provides an overall description of devices to perform this technique while discussing the nanotechnology behind its mechanism of action, its potential advantages, and clinical implications. By exploring current research and advancements, we aim to provide a comprehensive understanding of this novel method, addressing the critical questions of what it is, how it works, why it is needed, and when it should be applied. Special attention is given to patient selection and the optimal timing for therapy initiation in Alzheimer’s, coinciding with the peak accumulation of amyloid oligomers in the early stages. Potential limitations and alternative targets beyond beta-amyloid and future perspectives for immunoselective nanopheresis are also described.

## 1. Introduction

### 1.1. Overview of Alzheimer’s Disease

Alzheimer’s disease (AD) is the most common form of dementia, characterized by progressive neurodegeneration that leads to cognitive decline, memory loss, and eventual loss of independence in affected individuals.

#### 1.1.1. Prevalence and Risk Factors

AD is a global health concern, affecting approximately 50 million people worldwide, with numbers expected to triple by 2050, due to aging populations. Prevalence increases dramatically with age, with estimates suggesting that nearly one-third of individuals over the age of 85 have AD or another form of dementia. AD accounts for 60–80% of dementia cases worldwide, affecting millions of people, particularly those over the age of 65 [1].

1. Age: the greatest risk factor for Alzheimer’s is age, with most cases diagnosed after the age of 65. Early-onset Alzheimer’s (before age 65) is rarer, and often linked to genetic pathogenic variants.
2. Genetic Factors: several genetic risk factors have been identified. The apolipoprotein E (APOE) gene is the most well-known, particularly the APOE4 allele, which increases the risk of developing AD and is associated with earlier onset. Familial AD, a form of early-onset AD, is linked to pathogenic variants in the APP, PSEN1, and PSEN2 genes.
3. Lifestyle and environmental factors: cardiovascular health significantly impacts Alzheimer’s risk, with hypertension, hyperlipidemia, diabetes, obesity, and smoking increasing the likelihood of developing the disease. On the other hand, physical exercise, a healthy diet (such as the Mediterranean diet), and cognitive engagement are associated with lower risk. Sex Differences: women are disproportionately affected by AD, and while part of this is due to women living longer than men, biological and hormonal factors may also contribute to this increased risk.

#### 1.1.2. Pathophysiology

The hallmark pathophysiological features of AD include the accumulation of amyloid-beta (Aβ) plaques and neurofibrillary tangles composed of hyperphosphorylated tau protein. These deposits disrupt neuronal function and communication, leading to synaptic loss, neuronal death, and brain atrophy, especially in areas involved in memory and cognition like the hippocampus and cortex [2]. The pathogenic mechanism involves several key molecules and cellular changes, though their relative weight and interplay is a question of intense debate:

##### Amyloid-Beta

The “amyloid hypothesis” postulates that the overproduction or inadequate clearance of amyloid-beta peptides leads to the formation of plaques in the brain. These plaques disrupt cell communication and trigger immune responses, which leads to inflammation and further damage to neurons. Aβ is derived from amyloid precursor protein (APP), which is cleaved abnormally in AD, leading to the toxic Aβ40 and Aβ42 forms.

##### Tau

Tau proteins stabilize microtubules in healthy neurons. In Alzheimer’s, tau undergoes abnormal hyperphosphorylation, causing it to detach from microtubules and aggregate into neurofibrillary tangles. These tangles destabilize microtubules, leading to impaired intracellular transport and eventually cell death.

##### Neuroinflammation

In AD, chronic activation of microglia (the brain’s immune cells) and astrocytes contributes to neuroinflammation. While this immune response aims to clear toxic proteins, excessive inflammation exacerbates neuronal damage.

##### Synaptic Dysfunction and Neuronal Death

Synaptic loss is an early feature of AD, preceding neuron loss. As the disease progresses, widespread neuronal death occurs, leading to brain atrophy. The loss of cholinergic neurons, in particular, contributes to cognitive deficits, and has led to the development of acetylcholinesterase inhibitors as a symptomatic treatment.

#### 1.1.3. Treatment

There is no cure for AD, and current treatments primarily address symptoms. Cholinesterase inhibitors (donepezil, rivastigmine, and galantamine) and NMDA receptor antagonists (memantine) can modestly improve cognitive function, but do not halt disease progression. Emerging therapies target disease-modifying mechanisms, including amyloid-beta and tau. Anti-amyloid antibodies such as Aducanumab and Lecanemab have been approved, though their clinically relevant efficacy remains controversial. Moreover, these therapies are costly, and are associated with side effects, while penetrance to the CNS is limited.

### 1.2. CNS-Targeted Drug Delivery

Administering drugs to the Central Nervous System (CNS) poses significant challenges, primarily due to the restricted penetration of drugs through the Blood–Brain Barrier (BBB). The BBB effectively restricts the entry of various substances, including therapeutic agents, into the CNS [3,4]. Small molecule diffusion through the BBB is often limited, with passage influenced by the molecule’s size and structure. Molecules with a molecular weight (MW) exceeding 450 Daltons or those with polar functional groups forming more than seven hydrogen bonds typically exhibit low transport across the BBB unless facilitated by carrier-mediated transport systems [5,6]. Conversely, molecules with a MW below 450 Daltons and fewer hydrogen bonds may cross the BBB more readily, provided they are not substrates for active efflux transporters [7].

In pathological conditions such as stroke or cancer, structural and functional alterations in the BBB can increase permeability, allowing immune cells and other substances to pass into the CNS [8]. Physical interventions, such as hyperosmotic infusions or focused ultrasound, can temporarily disrupt the BBB to facilitate drug delivery [9]. However, in chronic conditions like neurodegenerative diseases, BBB dysfunction is exacerbated by chronic inflammation, oxidative stress, and pathological protein accumulation, further impeding drug delivery [10,11].

Innovative drug delivery technologies are crucial for overcoming these barriers and achieving CNS-targeted drug delivery. Nanomedicine has advanced with nanoparticle-based systems, including exosomes, liposomes, and micelles, which can encapsulate drugs such as monoclonal antibodies (mAbs) and show potential for effective CNS delivery [12]. For instance, lipid-based nanosystems, including nanoemulsions and nanostructured lipid carriers have been proposed for nose-to-brain delivery. These formulations have characteristics that facilitate drug transport directly to the brain, minimizing side effects and maximizing therapeutic benefits [13,14,15].

Invasive techniques for CNS drug delivery, such as implantable devices accessing the cerebrospinal fluid (CSF) through intrathecal, intraventricular, or intraparenchymal methods, have been utilized for some time. Devices like Omaya reservoirs and intrathecal pumps have been used clinically for decades, though recent developments have been limited. These devices have shown efficacy in treating various CNS conditions, including pain, spasticity, and CNS neoplasms, but are associated with complications such as infections, granuloma formation, and CSF flow obstruction [16,17,18]. In intrathecal pseudodelivery devices, the drug is encapsulated in a porous capsule in communication with the CSF. Here, the drug is not released into the CSF; rather, the CSF interacts with the capsule through its nanoporous walls, allowing the drug to act on its target within the capsule itself. This method allows for localized treatment, while avoiding systemic distribution of the drug, potentially reducing side effects and enhancing therapeutic efficacy for CNS conditions [19].

### 1.3. Nanofiltration and Nanoporous Membranes in Biomedical Applications

Apheresis is a medical technology where a biofluid is passed through an apparatus that separates a specific constituent and returns the remainder to circulation. Nanofiltration, a pressure-driven membrane separation technology, has gained significant attention in biomedicine and drug delivery, due to its ability to selectively separate molecules based on size and charge. The pore size of nanofiltration membranes typically ranges from 1 to 10 nm, enabling efficient separation of small organic molecules, multivalent ions, and proteins from larger components [20,21].

Nanoporous membranes have emerged as critical tools in medicine, particularly in fluid filtration and controlled drug delivery. These membranes, characterized by their high surface area and tunable pore sizes, offer exceptional selectivity and efficiency in filtering biological fluids, including proteins, enzymes, and antibodies, ensuring high purity and activity [22,23,24,25]. Nanoporous membranes possess selective molecular permeability, based on the size of molecules and other physical properties such as tridimensional structure and electrostatic charge. In medical applications, nanoporous membranes are used for separating proteins, viruses, and other biological molecules based on size. Nanofiltration has become a powerful tool in biomedicine, particularly for applications requiring precise separation and purification of biomolecules. Its ability to filter molecules selectively without harsh chemicals or extreme conditions makes it attractive for the pharmaceutical and biotechnology industries.

By controlling the molecular weight cut-off and surface properties of nanofiltration membranes, researchers can design drug carriers that precisely control the release of therapeutic agents from a reservoir to the target tissue or fluid. This precision enhances drug efficacy, while minimizing side effects. Nanoporous membranes can also be engineered to provide sustained, long-term drug release or to respond to specific stimuli, such as changes in pH or temperature, to release drugs on demand. This control helps maintain drug concentrations within the therapeutic window, reducing side effects and improving efficacy. Alternatively, nanoporous membranes can prevent the release of large compounds, while allowing fluids with soluble, smaller molecules to access the reservoir [19]. Nanoporous materials like mesoporous silica and anodized alumina have been extensively studied for these applications. Their biocompatibility and surface functionalization capabilities make them ideal for creating personalized and responsive drug delivery systems tailored to individual patient needs. Specifically in neurodegenerative diseases, nanoporous alumina membranes created through electrochemical anodizing feature highly uniform pore sizes, which can be adjusted to target specific molecules. This precision allows effective filtration of unwanted components from biological fluids, making them valuable for treating conditions like AD by filtering proteins that may contribute to neurodegeneration [26] or delivering drugs [27].

### 1.4. Targeting Soluble Amyloid-β Oligomers (SAβOs) in Alzheimer’s Disease

Neurodegenerative diseases impact millions worldwide, and are characterized by progressive dysfunction and neuronal loss. The specific etiologies of the sporadic forms of these conditions remain unclear, but many share a common feature: the accumulation of one or more proteins, often referred to as proteinopathies. In 1984, Glenner and Wong identified Aβ as a central component of extracellular amyloid plaques in AD [28,29]. Aβ has since been considered a driver of Alzheimer’s pathological processes, and the “amyloid cascade hypothesis” has become a leading theory of AD pathogenesis [29]. Despite significant research efforts, targeting Aβ has faced challenges, with many clinical trials failing to show success [30]. Recent research suggests that soluble amyloid-β oligomers (SAβOs), which accumulate early before amyloid plaque formation, may be a more relevant target [31,32,33,34,35]. SAβOs impair memory and cognitive function independently of amyloid plaques. Clinical evidence indicates that targeting SAβOs could be an effective approach to slow or halt disease progression: (1) agents targeting soluble Aβ oligomers show clinical efficacy; (2) amyloid plaque clearance does not correlate with clinical improvements; (3) agents targeting Aβ monomers or plaques fail to show significant effects; and (4) efficacy is higher in carriers of the ε4 allele of apolipoprotein E (APOE4), who have higher brain Aβ oligomer concentrations. Thus, inhibiting Aβ neurotoxicity appears to reduce tau pathology, suggesting a sequence where amyloid toxicity leads to increased tau formation and deposition [36].

#### 1.4.1. Strategies for Clearing SAβOs from the Periphery

The BBB not only acts as a physical barrier, but also regulates the movement of substances between the blood and the brain, maintaining crucial molecular balances for brain function and immune activity [34]. Low-density lipoprotein receptor-related protein 1 (LRP1) and RAGE are key proteins involved in Aβ transport across the BBB. LRP1 facilitates Aβ removal from the brain to the peripheral system, while RAGE aids Aβ entry into the brain, maintaining an equilibrium of Aβ levels between brain and peripheral blood. The “peripheral sink hypothesis” suggests that clearing Aβ in the periphery could enhance Aβ clearance from the CNS [37]. Several therapeutic strategies based on this hypothesis have been developed:

##### Plasmapheresis

Plasmapheresis is a therapeutic intervention that involves extracorporeal removal, return, or exchange of blood plasma or components. In plasma exchange, the removed plasma is substituted with an equal volume of plasma or colloid solutions. In AD treatment, plasmapheresis and plasma albumin replacement (i.e., the AMBAR study) aim to remove SAβOs from the plasma by replacing albumin, which binds to SAβOs [38,39,40]. This approach is based on the hypothesis that albumin replacement shifts Aβ forms from CSF to plasma, promoting natural degradation of Aβ. Clinical trials have observed a reduction in plasma Aβ and cognitive decline in moderate AD patients [41]. However, most Aβ cleared at the plasma level is likely peripheral, rather than cerebral [39]. Despite this, plasma exchange also reduces proinflammatory elements, contributing to decreased cognitive decline and gaining support from the American Society of Apheresis [40,41,42].

##### Dialysis

Dialysis, including hemodialysis and peritoneal dialysis, removes waste products and metabolites from the blood, potentially reducing Aβ levels [43,44,45,46,47]. Jin et al. found that peritoneal dialysis specifically decreased SAβOs levels in both blood and brain ISF [45]. This effect might be due to dialysis facilitating Aβ movement from the brain to the blood, possibly by increasing LRP1 levels and decreasing RAGE levels, thus reducing neuroinflammation [46].

##### Immunotherapy

Monoclonal antibodies (mAbs) have shown promise in targeting pathological proteins in neurodegenerative diseases. In AD, immunotherapy is a sophisticated strategy to combat Aβ pathology. mAbs targeted against Aβ are designed to reduce Aβ plaque formation and lower Aβ levels [48,49,50]. A list of the most relevant passive immunotherapies for AD is shown in Table 1. Currently approved Aβ-targeting mAbs by the FDA include Aducanumab and Lecanemab [51,52,53,54,55]. These antibodies act through different mechanisms: Aducanumab targets aggregated Aβ plaques, enhancing their removal, while Lecanemab targets soluble forms of Aβ, and has shown effects in reducing amyloid plaque burden and slowing cognitive decline (Table 1). The clinical effectiveness of these antibodies varies, but they represent a promising direction for targeted therapy in AD.

#### 1.4.2. Strategies for Clearing SAβOs Directly from the CNS

Soluble proteins in the ISF, like soluble Aβ, can be removed from the CSF, known as the CSF-sink therapeutic strategy [56,57,58]. For instance, as Aβ is in constant equilibrium between the ISF and the CSF, several research studies have suggested a method to address the neuropathology of AD by directly eliminating Aβ from the CSF. As the CSF-ISF barrier is more permeable than the BBB, CSF filtration (liquorpheresis) is expected to have a stronger effect on clearing CNS deposits compared to plasmapheresis. To date, only a few studies have attempted to decrease target molecules from the CSF using physical methods [59]. Extracorporeal liquorpheresis involves using extracorporeal systems to filter CSF and remove molecules from it. The filtered fluid is then returned to the ventricular system or the subarachnoid space. This method has been previously explored for treating autoimmune and neurodegenerative diseases and used as a potential treatment for CNS infections and subarachnoid hemorrhages or leptomeningeal disease [60,61,62,63,64,65,66,67].

Similar to the mechanism of action in the AMBAR study based on replacing amyloid-binding proteins, some researchers have proposed the replacement of CSF Aβ-binding molecules, such as albumin or transthyretin [68]. However, the infusion of these proteins carries the potential risk of elevated intracranial pressure due to albumin’s osmotic properties. Achieving a precise osmotic balance to maintain a neutral pressure effect carries additional challenges. 

## 2. What Is Intrathecal Immunoselective Nanopheresis?

Intrathecal immunoselective nanopheresis is an experimental technique designed to selectively clear target molecules from CSF, using nanotechnology combined with immunological targeting. This method employs mAbs to provide molecular selectivity, making it a promising approach for filtering specific proteins or pathological agents from the CSF.

### 2.1. States of mAbs

Immunoselective nanopheresis leverages the specificity of mAbs to target and bind to particular molecules within a fluid. The system involves a device that incorporates a nanoporous membrane, which acts as a selective filter. There are two main states or forms of mAb application in this system:

4. **Immobilized mAbs:** in this approach, mAbs are fixed onto a substrate within the device. The nanoporous membrane allows the target molecules in the fluid to pass through and interact with the immobilized mAbs, facilitating their removal from the fluid.
5. **Soluble mAbs:** alternatively, mAbs can be dissolved in a solution and contained within a capsule fitted with a nanoporous membrane. This membrane prevents the mAbs from escaping while permitting target molecules to enter the capsule. Once inside, the mAbs interact with the target molecules, effectively capturing and removing them from the fluid [19].

### 2.2. Application in Intrathecal Pseudodelivery

**Figure 1:**
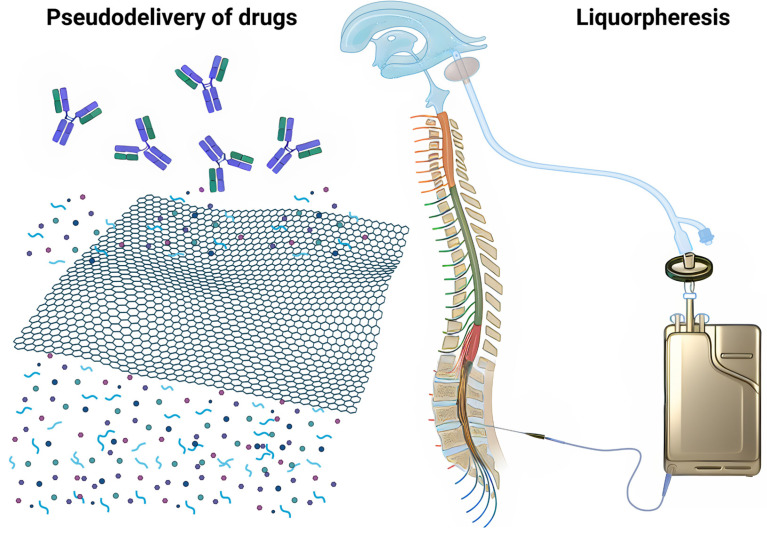
Conceptual illustration of intrathecal immunoselective nanopheresis. This figure depicts the use of nanoporous membranes in combination with soluble mAbs to selectively filter target low size molecules from the CSF. Immunoselective nanopheresis can both be considered a type of drug pseudodelivery, where the compounds are monoclonal antibodies that provide the target-selectivity (on the **left**); and a way to achieve CSF filtration (liquorpheresis) (on the **right**).

Conceptually, the process can be described as any of the following therapeutic strategies (Figure 1):

- **Intrathecal Pseudodelivery:** in this context, the pseudodelivery refers to the device’s function in filtering target molecules from the CSF without releasing the mAbs themselves into the fluid. The nanoporous membrane and the presence of soluble mAbs enable selective interaction with the target molecules, facilitating their removal [67].
- **Liquorpheresis:** this process is a type of CSF filtration or liquorpheresis, where the goal is to purify the CSF by removing unwanted substances, such as amyloid-β oligomers or other pathological agents, while leaving essential components intact.

### 2.3. Advantages and Potential

The use of immunoselective nanopheresis offers several advantages:

- **Target Specificity:** the combination of nanoporous membranes and mAbs allows for precise targeting of specific molecules, reducing off-target effects and increasing the efficiency of the filtration process.
- **Reduced Side Effects:** by focusing on the removal of specific pathological agents from the CSF, this method minimizes potential systemic side effects associated with broader therapeutic interventions.
- **Enhanced Therapeutic Outcomes:** improved targeting and selective clearance of harmful molecules can lead to better management of neurological diseases, potentially slowing disease progression and improving patient outcomes.

## 3. How Does Intrathecal Immunoselective Nanopheresis with Soluble mAbs Work?

**Figure 2:**
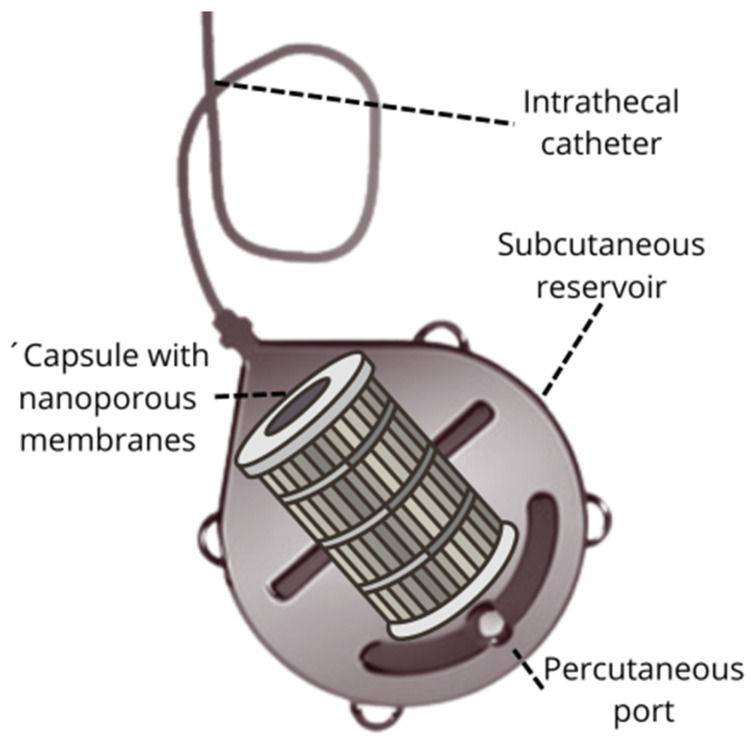
Key components in an intrathecal immunoselective nanopheresis device for soluble mAbs: the intrathecal catheter and the subcutaneous reservoir with a port for percutaneous access and a capsule with nanoporous membranes for selective molecular permeability.

Intrathecal immunoselective nanopheresis (ISN) involves an advanced implantable device designed to selectively filter pathological molecules from cerebrospinal fluid (CSF) using soluble monoclonal antibodies (mAbs). This technique integrates two key components: a subcutaneous reservoir with nanoporous membranes and a system of intrathecal catheters (Figure 2).

### 3.1. Subcutaneous Reservoir with Nanoporous Membranes

The core of the ISN system is an implantable device with a subcutaneous reservoir that houses nanoporous membranes. These membranes are engineered with specific pore sizes to selectively filter out target molecules from the CSF. The design of the nanoporous membranes can be customized to address various pathological targets, depending on the disease being treated [19].

Key features of the subcutaneous reservoir include the following:

- **Nanoporous Membranes:** these membranes are designed to capture pathological molecules based on their size and characteristics. The ability to tailor pore sizes allows for precise targeting of different molecules, enhancing the effectiveness of the filtration process.
- **Subcutaneous Placement:** the reservoir is implanted beneath the skin, providing a minimally invasive approach for continuous therapy. Its placement ensures easy access for maintenance and replacement procedures.

### 3.2. Systems for CSF Flow

The subcutaneous reservoir is connected to one or more intrathecal catheters, which are placed directly into the CSF space to facilitate efficient fluid flow and molecule clearance. The system for CSF flow can be managed in several ways:

- **Electromechanical Pumps:** traditional systems use electromechanical pumps to regulate CSF flow. These pumps, similar to those used in intrathecal drug delivery systems, can be programmed to achieve the desired flow rate. However, they are limited by battery life, weight, and MRI compatibility issues.
- **Smart Catheters:** an alternative approach involves the use of “smart catheters” that can manage CSF flow without the need for electromechanical pumps. These catheters are designed to ensure continuous fluid flow, while avoiding the limitations of traditional pump systems.

As CSF flows through the device, the nanoporous membranes capture pathological molecules, effectively removing them from circulation. This selective filtration reduces the concentration of harmful substances in the CSF, which can

- **Alleviate Symptoms:** reduction in the concentration of target molecules can help alleviate symptoms associated with various neurological conditions [61].
- **Slow Disease Progression:** by removing pathological molecules, the system may slow the progression of neurodegenerative diseases.

### 3.3. Preliminary and Ongoing Research

Preliminary studies using miniaturized prototypes in transgenic mice models of AD have demonstrated the feasibility and effectiveness of this therapy. Results include the following:

- **Reduction in Amyloid Plaques:** significant reduction in the number of amyloid plaques in the brain [69,70].
- **Restoration of BBB Function:** improvement in the integrity and function of the BBB [70].
- **Enhanced Cellular and Fluidic Clearance:** restoration of the brain’s natural clearance mechanisms [59,68,69,70].

Moreover, percutaneous replacement of mAbs, once they become saturated, allows for long-term therapy with a manageable regimen. Encouraging results in animal models have led to the development of human prototypes, which have been successfully implanted in large animals such as pigs [69].

## 4. Why Is Intrathecal Immunoselective Nanopheresis Needed?

Intrathecal immunoselective nanopheresis provides several advantages over traditional systemic or intrathecal immunotherapies, addressing specific challenges associated with these methods. The key reasons for its need include the following:

### 4.1. Enhanced Targe-Molecule Clearance

ISN offers a superior rate of target-molecule clearance from cerebrospinal fluid (CSF) compared to plasma. This is primarily due to the higher permeability of the CSF–brain barrier, which allows for more efficient removal of pathological molecules from the CSF. By focusing on the CSF, ISN can achieve higher concentrations of clearance for target molecules than systemic treatments, which need to traverse multiple barriers before reaching the central nervous system (CNS) [59,69,70].

### 4.2. Reduced Risk of Systemic Side Effects

Systemic therapies often come with risks such as allergic reactions and amyloid-related imaging abnormalities (ARIAs) [71]. Intrathecal immunoselective nanopheresis minimizes these risks through the use of nanoporous membranes that isolate the therapeutic agents within the CSF, preventing systemic exposure. This immunoisolation reduces the likelihood of adverse reactions and enhances patient safety [59].

### 4.3. Continuous and Efficient Treatment

The implantable nature of the intrathecal immunoselective nanopheresis device allows for continuous ambulatory treatment. This contrasts with intermittent systemic therapies, which may be less efficient and require frequent administration. Continuous treatment with intrathecal immunoselective nanopheresis can lead to more significant and sustained clearance of pathological molecules, potentially resulting in better therapeutic outcomes [59,69,70,72].

### 4.4. Integration with Other Therapeutic Approaches

ISN can be combined with other therapeutic strategies to enhance its effectiveness. For example, some oral therapeutics aim to disintegrate high-molecular-weight aggregates of amyloid plaques into soluble oligomers. An example is levodopa, which enhances brain neprilysin activity to achieve this effect [73]. However, these newly formed soluble oligomers need to be removed to prevent re-nucleation and reaggregation. Intrathecal immunoselective nanopheresis provides a means to efficiently clear these soluble oligomers from the CSF.

### 4.5. Flexibility and Personalization

The flexibility of the immunoselective nanopheresis system allows it to target various molecules beyond Aβ, including those involved in enzymatic dysfunction and inflammation. This adaptability makes it possible to tailor treatments based on the specific proteins and pathological factors present in a patient at different stages of disease progression. For instance, immunoselective nanopheresis could be combined with mAbs targeting other proteins, such as Tau or inflammation-related markers, to provide a truly personalized medicine approach.

## 5. When Should Intrathecal Immunoselective Nanopheresis Be Applied?

### 5.1. Disease Stage

**Figure 3:**
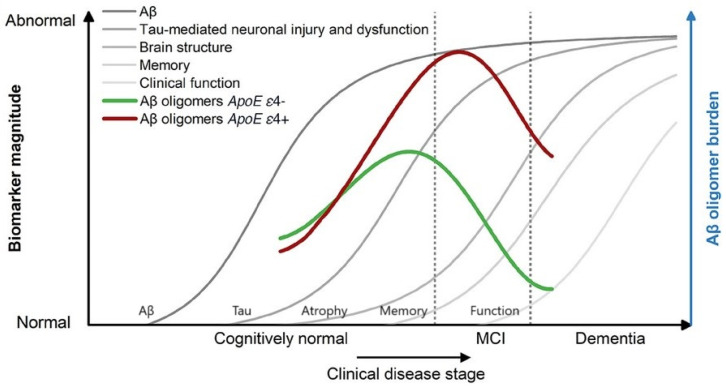
Hypothetical changes in Aβ oligomers during disease progression are transferred to the model of AD biomarker changes, according to Jack et al. A peak in sAβOs takes place in the early symptomatic or presymptomatic stage. Apolipoprotein E (APOE) gene ε4 allele carriers show higher oligomer concentrations, with a peak at a more advanced disease stage, but still in the early stages of the disease. Image from Blomeke [36], modified after Jack et al. [74].

Intrathecal immunoselective nanopheresis against sAβOs is expected to be particularly beneficial during the early stages of AD. Research indicates that the accumulation of sAβOs in the CSF peaks early in the AD continuum, notably during mild cognitive impairment (MCI) and subjective cognitive decline (SCD) (Figure 3) [35]. This timing represents a critical window for intervention, as targeting Aβ oligomers at these early stages, before tau pathology becomes prominent, may maximize therapeutic efficacy [74]. In contrast, levels of Aβ oligomers tend to decrease in advanced stages of the disease (A+T+), possibly due to altered clearing mechanisms and the progression of other pathological processes [74]. Therefore, initiating intrathecal immunoselective nanopheresis early in the disease course may offer the most significant benefits.

### 5.2. Patient Selection

While Intrathecal immunoselective nanopheresis has potential benefits, patient selection is crucial for its successful application. Several factors must be considered:

#### 5.2.1. Disease Type and Stage

Not all patients with AD are suitable candidates for intrathecal immunoselective nanopheresis. The choice of patients should be based on the specific disease stage and overall health condition. The therapy is most appropriate for individuals in the early stages of AD, where there is a high burden of sAβOs.

#### 5.2.2. Genetic Factors

ApoE4 carriers are particularly relevant for this therapy. ApoE4 is a well-established risk factor for AD, associated with a higher burden of Aβ plaques and an increased risk of adverse effects from systemic immunotherapies. ApoE4 carriers are more prone to amyloid-related imaging abnormalities (ARIA), such as cerebral edema and microhemorrhages, due to potential differences in blood–brain barrier permeability and immune response mechanisms [71]. While intrathecal immunoselective nanopheresis offers a promising alternative by providing localized, continuous treatment with reduced systemic exposure, it is essential to consider these patients’ unique needs.

#### 5.2.3. Overall Health

Candidates should be assessed for their ability to undergo minimally invasive surgery required for device implantation. While the procedure is generally less invasive, patient health and surgical risks must be evaluated.

## 6. Limitations, Alternative Targets, and Future Perspectives

### 6.1. Limitations

Despite its advanced design and potential, intrathecal immunoselective nanopheresis faces several limitations, compared to other therapeutic approaches in AD.

#### 6.1.1. Target Limitations

While intrathecal immunoselective nanopheresis utilizes monoclonal antibodies to selectively target pathological proteins such as Aβ, its ability to remove other neurotoxic species involved in AD pathology is limited to those that are soluble and present in the CSF [21]. Insoluble molecules or those that are present intracellularly only would be beyond the scope of intrathecal immunoselective nanopheresis. This restricts the scope of intrathecal immunoselective nanopheresis compared to more holistic interventions such as pharmacological treatments, which target multiple pathways simultaneously, although a combination of monoclonal antibodies for two or more targets is feasible [71,75].

#### 6.1.2. Invasiveness and Complication Risks

Intrathecal immunoselective nanopheresis requires the surgical implantation of a reservoir and catheter system, which introduces risks of infection, mechanical failure, or catheter-related complications such as obstruction or dislodgement. In contrast, less invasive therapeutic options, such as intravenous monoclonal antibodies or oral small-molecule drugs, offer safer profiles with fewer procedural risks. These minimally invasive options can achieve systemic delivery of therapeutic agents without the need for surgical procedures.

#### 6.1.3. Challenges with Long-Term Efficacy

Intrathecal immunoselective nanopheresis relies on sustained device functionality and continuous filtration of pathological proteins. While percutaneous redosing to the subcutaneous reservoir allows convenient long therapy, the filtration efficiency of membranes may decrease over time, requiring device maintenance or replacement, which poses additional risks [76]. Moreover, the long-term efficacy of intrathecal immunoselective nanopheresis in slowing or halting disease progression has not yet been fully demonstrated in clinical settings, unlike other therapies that have shown sustained benefits over extended periods in clinical trials.

#### 6.1.4. Cost and Accessibility

The complexity of intrathecal immunoselective nanopheresis, including its need for surgical implantation, long-term monitoring, and maintenance, likely incurs significant costs. This could limit its accessibility, particularly when compared to more conventional pharmacological approaches that, although potentially expensive, are more widely available and can be administered without surgical intervention.

### 6.2. Alternative Targets

The role of amyloid-beta in Alzheimer’s disease has long been a subject of debate. While amyloid plaques are a defining pathological feature of the disease, interventions aimed at clearing amyloid-beta from the brain have yielded inconsistent results in clinical trials, raising questions about the precise role of amyloid-beta in disease progression [77,78]. Identifying effective targets within the cerebrospinal fluid that could dissolve amyloid plaques remains a complex challenge. Current research has suggested several mechanisms through which modulation of amyloid metabolism in the cerebrospinal fluid could aid in the dissolution of amyloid plaques. Several promising approaches focus on modulating metabolism, including apolipoprotein E isoforms, beta-secretase (BACE1) inhibitors, amyloid-binding proteins, and amyloid-degrading enzymes. Targeting these pathways could help reduce amyloid burden and potentially mitigate disease progression.

#### 6.2.1. Apolipoprotein E (ApoE) Isoforms

Apolipoprotein E is a critical regulator of amyloid metabolism in the brain, and its isoforms have distinct effects on amyloid-beta aggregation and clearance. The apolipoprotein E4 isoform, in particular, is strongly associated with an increased risk of AD, as it promotes amyloid-beta aggregation and impairs clearance mechanisms. Studies have shown that reducing apolipoprotein E4 levels or blocking its interaction with amyloid-beta in the cerebrospinal fluid could promote amyloid clearance, while the apolipoprotein E2 and apolipoprotein E3 isoforms play protective roles by facilitating amyloid-beta degradation [79,80].

#### 6.2.2. Soluble Amyloid Precursor Protein Beta (sAPPβ)

sAPPβ is produced through the cleavage of amyloid precursor protein by beta-secretase, a key enzyme in the amyloidogenic pathway. This leads to the production of amyloidogenic amyloid-beta peptides. Theoretically, by targeting soluble amyloid precursor protein beta, it may be possible to reduce amyloid-beta production at its source. 

#### 6.2.3. Beta-Secretase (BACE) Inhibitors

Beta-secretase is responsible for the initial cleavage of amyloid precursor protein, which leads to the production of amyloid-beta. Inhibiting this enzyme could prevent the generation of amyloid-beta and consequently reduce plaque formation. While some beta-secretase inhibitors have shown promise in reducing amyloid-beta levels in preclinical models, clinical trials have been less conclusive, highlighting the need to further refine this approach [81,82].

#### 6.2.4. Amyloid-Binding Proteins

Certain proteins in the CSF, such as albumin, transthyretin, clusterin and components of the complement system, bind to amyloid-beta and facilitate its aggregation into plaques. Targeting these amyloid-binding proteins to reduce their interaction with amyloid-beta could limit plaque propagation and assist in plaque clearance [68].

#### 6.2.5. Pro-Inflammatory Cytokines

Neuroinflammation plays a significant role in Alzheimer’s disease pathology. Elevated levels of pro-inflammatory cytokines, such as interleukin-1β and tumor necrosis factor-alpha, have been observed in AD patients. Chronic inflammation exacerbates amyloid pathology by promoting amyloid-beta aggregation. Reducing inflammation through cytokine inhibition may create a more favorable environment for amyloid clearance [83].

#### 6.2.6. Tau Protein

Though primarily associated with neurofibrillary tangles, tau pathology is also linked to amyloid-beta deposition. Reducing levels of hyperphosphorylated tau in the cerebrospinal fluid could indirectly decrease amyloid plaque formation and aid in plaque clearance. Tau-targeting therapies are currently under clinical and preclinical investigation, including several anti-tau monoclonal antibodies [84].

#### 6.2.7. Metalloproteins

Zinc and copper ions bind to amyloid-beta and promote its aggregation into plaques. Chelation of these metals from the cerebrospinal fluid has been proposed as a strategy to destabilize amyloid plaques and enhance their clearance. By interfering with metal-mediated aggregation, this approach could facilitate the dissolution of plaques [85].

### 6.3. Future Perspectives

The future of intrathecal immunoselective nanopheresis holds significant promise for advancing the treatment of AD and other neurodegenerative disorders. As research continues to optimize its technology, expand its molecular targets, and explore its role in combination therapies and early intervention, intrathecal immunoselective nanopheresis could become an integral component of personalized and preventive approaches to neurodegenerative disease management.

Future strategies could involve combining intrathecal immunoselective nanopheresis with other therapeutic modalities, such as anti-amyloid antibodies, tau-targeting drugs, or neuroprotective agents. This combined approach may increase overall treatment efficacy by simultaneously reducing pathological burden and preventing further neurodegeneration.

### Technological Optimization

Advancements in nanotechnology and device engineering will be critical for refining the intrathecal immunoselective nanopheresis system. Future iterations of intrathecal immunoselective nanopheresis could feature enhanced nanoporous membranes with higher selectivity, improved durability, and greater filtering capacity. This can be achieved by means of coating membranes with advanced materials. Additionally, reducing the invasiveness of the implantation process and developing more user-friendly maintenance protocols will be important for increasing patient acceptance and minimizing risks associated with long-term device use.

The future of intrathecal immunoselective nanopheresis may lie in its potential for tailoring treatments to individual patients. By analyzing a patient’s specific molecular profile, intrathecal immunoselective nanopheresis could be customized to remove the exact pathological proteins most relevant to their disease. This personalized approach would ensure that patients receive the most effective intervention, based on their unique disease trajectory, optimizing therapeutic outcomes.

## 7. Conclusions

Intrathecal immunoselective nanopheresis presents a promising and targeted approach for treating AD and other neurodegenerative disorders, by selectively removing pathological molecules from the CSF. This innovative method offers advantages over existing therapies, including the precise clearance of harmful proteins, potentially reducing off-target effects and improving treatment efficacy. However, ISN also faces significant challenges, such as invasiveness, long-term efficacy, and cost-effectiveness. Further research, clinical trials, and refinement are necessary to validate its safety and effectiveness and to determine its place in the broader therapeutic landscape. Early intervention during key periods of protein accumulation may be critical for achieving optimal therapeutic outcomes. The continued development of immunoselective nanopheresis, along with the exploration of alternative targets, will be essential for its successful integration into clinical practice.

## 8. Patents

This therapeutic strategy is based on International Patent No. WO2019/158791A1.

## Figures and Tables

**Table 1:** Passive immunotherapies explored for AD, including their target populations, mechanisms of action and main findings [51,52,53,54,55].

Agent	Target Population	Mechanism of Action	Main Findings
Aducanumab	Early-stage Alzheimer’s disease (prodromal or mild AD)	Monoclonal antibody targeting amyloid-beta to reduce amyloid plaque deposition	Phase III showed reduction in amyloid plaques but mixed cognitive outcomes; approved by FDA, with controversial efficacy.
Lecanemab	Early-stage Alzheimer’s disease, preclinical AD with amyloid pathology	Monoclonal antibody that targets soluble amyloid-beta protofibrils for clearance	Phase III trial (CLARITY AD) showed a 27% reduction in cognitive decline and amyloid burden. Approved by FDA for early Alzheimer’s disease.
Donanemab	Early-stage Alzheimer’s disease, participants with amyloid plaques	Monoclonal antibody that specifically targets amyloid plaques in the brain	Phase III results showed a slowing of cognitive decline and significant reduction in amyloid plaques. Participants with intermediate tau levels showed the most benefit.
Solanezumab	Mild-to-moderate Alzheimer’s disease	Monoclonal antibody that targets soluble forms of amyloid-beta to reduce amyloid deposition	Phase III trials did not show significant cognitive benefits, despite plaque reduction.
Crenezumab	Mild-to-moderate Alzheimer’s disease and familial AD	Monoclonal antibody that targets multiple forms of amyloid-beta, including oligomers	Phase II trials showed plaque reduction but failed to show cognitive benefits in Phase III.
Gantenerumab	Mild-to-moderate Alzheimer’s disease	Monoclonal antibody targeting amyloid-beta to promote clearance of plaques	Failed to meet primary outcomes in Phase III, despite reduction in amyloid plaques.
